# Bactericidal Effects of 405 nm Light Exposure Demonstrated by Inactivation of *Escherichia, Salmonella, Shigella, Listeria, and Mycobacterium* Species in Liquid Suspensions and on Exposed Surfaces

**DOI:** 10.1100/2012/137805

**Published:** 2012-04-01

**Authors:** Lynne E. Murdoch, Michelle Maclean, Endarko Endarko, Scott J. MacGregor, John G. Anderson

**Affiliations:** The Robertson Trust Laboratory for Electronic Sterilisation Technologies, Department of Electronic and Electrical Engineering, University of Strathclyde-Glasgow, Glasgow G1 1XW, UK

## Abstract

The bactericidal effect of 405 nm light was investigated on taxonomically diverse bacterial pathogens from the genera *Salmonella, Shigella, Escherichia, Listeria, and Mycobacterium*. High-intensity 405 nm light, generated from an array of 405-nm light-emitting diodes (LEDs), was used to inactivate bacteria in liquid suspension and on exposed surfaces. *L. monocytogenes* was most readily inactivated in suspension, whereas *S. enterica* was most resistant. In surface exposure tests, *L. monocytogenes* was more susceptible than Gram-negative enteric bacteria to 405 nm light when exposed on an agar surface but interestingly less susceptible than *S. enterica* after drying onto PVC and acrylic surfaces. The study findings, that 405 nm light inactivates diverse types of bacteria in liquids and on surfaces, in addition to the safety advantages of this visible (non-UV wavelength) light, indicate the potential of this technology for a range of decontamination applications.

## 1. Introduction

Despite enormous investments in public health research, bacterial pathogens transmitted in food, water, and from other environmental sources remain a major cause of illness in both the developed and developing world. Examples of such ubiquitous pathogens include enteric Gram-negative bacteria such as *Salmonella, Escherichia*, and *Shigella* which continue to cause significant diarrhoeal infections worldwide [1]. The foodborne pathogen *Listeria monocytogenes *also, has significant impact on health statistics through its propensity for causing serious illness in the immunocompromised [2]. Actinobacteria from the *Mycobacterium *genus are also a major cause of human morbidity and mortality and pathogens from the *Mycobacterium tuberculosis* complex such as *M. bovis* and *M. tuberculosis* remain amongst the most serious causes of infective disease worldwide [3]. 

Whilst many traditional decontamination methods give sterling service, they are not without limitations. Problems associated with product/material damage related to the use of physical methods and development of microbial resistance as well as the formation and persistence of potentially harmful residues are longstanding issues related to the use of chemical disinfectants such as sodium hypochlorite, ozone, and H_2_O_2_ [4–7]. Mycobacteria have innate resistance to chemical decontamination as they possess an unusual cell envelope containing peptidoglycan, arabinoglycan, and mycolic acid. This restricts the passage of many chemicals/drugs across the cell envelope, contributing to the hardiness of this microorganism [8, 9].

In response to the ongoing challenges presented by established and emerging pathogens and to provide additional or alternative approaches to microbial control, considerable interest has developed concerning novel methods of disinfection and decontamination. 

Such alternative methods of decontamination include continuous and pulsed UV light, which has been shown to be highly germicidal. However, limitations such as poor transmissibility, degradative effects on materials, and potential carcinogenic effects in humans mean that there are restrictions in its use [10–13]. A safe, non-UV, light-based decontamination technology termed high-intensity narrow-spectrum (HINS) light has been recently described. HINS light of 405 nm stimulates endogenous microbial porphyrin molecules to produce oxidising reactive oxygen species (ROS), predominantly singlet oxygen (^1^O_2_) that damages cells leading to microbial death [14–16]. Specifically 405 nm light has been shown to be capable of inactivating a range of predominantly nosocomial pathogens and also Gram negative food-related pathogens [17–22].

This study further investigates the inactivating effect of high-intensity 405 nm light exposure on taxonomically diverse bacterial pathogens. Inactivation data on three species of enteric facultatively anaerobic Gram negative bacilli (*Salmonella enterica*, *Shigella sonnei, *and* Escherichia coli*) were compared with the facultatively anaerobic Gram-positive coccobacillus *Listeria monocytogenes* and with the aerobic, acid fast Gram positive bacillus *Mycobacterium terrae*. The study also aimed to determine the effectiveness of 405 nm light for inactivating bacteria in both liquid suspensions and on exposed surfaces.

## 2. Materials and Methods

### 2.1. Microorganisms

The bacteria used in this study were *Salmonella enterica *serovar* enteritidis *NCTC 4444 (formerly known as *Salmonella enteritidis*),* Shigella sonnei *NCTC 12984, *Escherichia coli* serotype O157:H7 NCTC 12900, *Listeria monocytogenes *NCTC 11994, and *Mycobacterium terrae* LMG 10394. All cultures were obtained from the National Collection of Type Cultures, Colindale, UK, except *M. terrae*, which was obtained from the Laboratorium voor Microbiologie, Universiteit Gent, Belgium. *M*. *terrae* was chosen as a safe, comparative, surrogate microorganism for the highly pathogenic *M. tuberculosis*. *S. sonnei, S. enterica, *and *E. coli* were inoculated into 100 mL Nutrient Broth, *L. monocytogenes* into 100 mL Tryptone Soya Broth (all Oxoid, Basingstoke, UK), and *M. terrae* into 100 mL of Middlebrook 7H9 Broth containing ADC enrichment media (Becton Dickinson and Company, NJ, USA). Broths were cultivated at 37°C for 18 hours under rotary conditions (120 rpm), with the exception of *M. terrae* which was cultivated at 37°C for 14 days, after which the purity was checked using Ziehl-Neelsen stain (Kinyoun method). After cultivation, broths were centrifuged at 3939× g for 10 minutes, and the resultant pellet resuspended in 100 mL phosphate-buffered saline (PBS) and serially diluted to give the appropriate starting population of 1-2 × 10^5^ CFU mL^–1^ for experimental use. 

### 2.2. 405 nm Light Exposure of Bacterial Suspensions

#### 2.2.1. Light Source

An indium-gallium-nitride 99-DIE light-emitting diode (LED) array (Opto Diode Corp, CA, USA) was used for exposure of bacterial suspensions. This array was 20 mm × 16 mm in size and had an emission at 405 nm with a bandwidth of 14 nm at full-width half maximum (FWHM). A cooling fan and heat sink were attached to the array to dissipate heat from the source and this also served to minimise any heat transfer to the sample. The LED array was mounted in a polyvinyl chloride (PVC) housing designed to fit a 12-well micro plate (NUNC, Roskilde, Denmark).

#### 2.2.2. Treatment of Bacterial Suspensions

For exposure of bacterial suspensions, the LED array was set in a fixed position 2 cm directly above a micro plate well, which held a 2 mL volume of bacterial suspension. To ensure that all bacteria in the suspension were uniformly suspended and exposed to the same dose over the exposure period, a small magnetic follower was incorporated in the well and the sample dish and LED array were positioned on a magnetic stirrer which permitted continuous stirring of the sample during light exposure. The LED array was powered by a DC power supply (0.5 A ± 0.05 and 11.2 V ± 0.2), giving an approximate irradiance (or power density) of 10 mW cm^–2^ at the surface of the bacterial suspension.

In order to quantitatively examine the inactivation process, it was necessary to account for any attenuation of the irradiance of the 405 nm light as it passed through a bacterial sample; attenuation is a result of light absorption and scattering. Attenuation by the samples used in the study was examined by measuring the irradiance as the light entered the surface of the sample and comparing that value with the irradiance immediately below the sample depth of 7 mm, after allowing for the transmission loss through the base of the sample dish. These measurements showed that, for samples containing bacterial populations of 10^7^ CFU mL^–1^ and less, no measurable attenuation, occurred, therefore; to ensure that no attenuation effects occurred, bacterial suspensions of 1-2 × 10^5^ CFU mL^–1^ were used in the current study.

#### 2.2.3. Dose Delivery Experiments

In order to establish if inactivation of bacteria was dose dependent, suspensions of *L. monocytogenes* of a population density of 1-2 × 10^5^ CFU mL^–1^ were exposed to 108 J cm^–2^ of 405 nm light applied using three different regimes. Three different light irradiances were used (10 mW cm^–2^, 20 mW cm^–2^, and 30 mW cm^–2^), and in order to keep the total dose constant in each case (i.e., 108 J cm^–2^), the sample exposure time was adjusted according to the equation:

(1) $$E^{\prime }=P^{\prime }t,$$

where *E*′ is the energy density (dose) in J cm^–2^, *P*′ is the irradiance (power density) in W cm^–2^, and *t* is the time in seconds. 

### 2.3. 405 nm Light Exposure of Bacteria Seeded onto Surfaces

#### 2.3.1. Light Source

**Figure 1:**
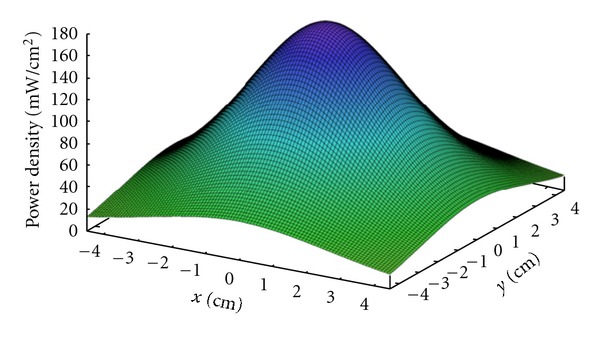
Three-dimensional model demonstrating the power density (mW cm^–2^) distribution of the emission from the 405 nm ENFIS QUATTRO LED array across a 9 cm agar plate.

An ENFIS QUATTRO Mini Air Cooled Light Engine (ENFIS Ltd, Swansea, UK) was used for exposure of bacteria seeded onto agar and inert surfaces as this source allowed more effective treatment of larger surface areas. This source was an array of 144 LEDs (40 mm × 40 mm in size) with emission at 405 nm (16 nm FWHM) and was powered by a 48 V power supply. The light engine had an integrated heat sink and cooling fan to minimise any heat buildup during experimentation. The optical distribution of the 405 nm LED array emission was measured across the length and breadth of (i) a 9 cm diameter agar plate and (ii) 6 × 4 cm coupons of inert materials (polyvinyl chloride and acrylic), in 0.5 cm increments, using a radiant power density meter calibrated at 405 nm. These measurements were then used to generate a three-dimensional model using Maxima Software release 5.2.2.1 (Figure 1). The average irradiance emitted by the light source was found to be 71 mW cm^–2^ across the agar plate and 110 mW cm^–2^ across the coupons, as calculated with Maxima Software release 5.2.2.1 using the Romberg Method:

(2) $$P=\frac{\iint_{-4.5}^{4.5}5^{\left(3+m\right)}\times E_{0}\times {\left(25+x^{2}+y^{2}\right)}^{-(3+m)/2}dx\;dy}{\pi \times r^{2}},$$

where *P* is the average irradiance/power density, *E*_0_ is the irradiance/power density at the plate centre, *m* is the Lambertian mode number and *x* and *y* are the cartesian coordinates.

#### 2.3.2. Quantitative Agar Surface Exposure Experiments

Bacterial suspensions of *S. enterica*, *E. coli*, *L. monocytogenes,* and *S. sonnei*, containing approximately 10^2^ CFU mL^–1^, were pipetted and spread onto the surface of 9 cm Tryptone Soya Agar (TSA) (Oxoid, Basingstoke, UK) plates, giving approximately 200–250 CFU/per plate, equivalent to 2.3-2.4 log_10_ CFU/plate. Seeded agar plates were then exposed to increasing durations of high-intensity 405 nm light. Nonexposed control plates were prepared for each 405 nm light-exposed sample. This test was not carried out for *M. terrae* as the organism does not grow on TSA and an accurate comparison could not have been drawn with different growth media.

#### 2.3.3. Qualitative Agar Surface Exposure Experiments

For a qualitative indication of the bactericidal effects of 405 nm light on surfaces, loopfuls of 10^6^ CFU mL^–1^bacterial suspensions of *S. enterica*, *E. coli*, *L. monocytogenes,* and *S. sonnei* were streaked in individual lines of length 6 cm (1 cm apart) onto the surface of a TSA plate. Half of each inoculum line (3 cm) was exposed to high-intensity 405 nm light, with the other half covered with aluminium foil to prevent light exposure. Plates were exposed to 15, 30, and 45 minutes of high-intensity 405 nm light, with an average irradiance of 71 mW cm^–2^across the plate. After exposure, plates were incubated at 37°C for 16 hours and photographs of each plate were taken. *M. terrae* was not included in this test due to its unique growth requirements.

#### 2.3.4. Inert Surface Exposure Experiments

Bacterial suspensions of *S. enterica* (as a representative Gram negative bacterium) and *L. monocytogenes* (as a representative Gram positive bacterium) containing approximately 10^5^ CFU mL^–1^ were loaded into a 6-jet Collison nebuliser (BGI Inc, MA, USA). Coupons of acrylic (6 cm × 4 cm) and polyvinyl chloride (PVC) (6 cm × 4 cm), pre-sterilised with 80% ethanol, were held at a distance of 3 cm from the nebuliser for 15 seconds allowing the aerosolised bacteria to deposit and immediately dry onto the surface of the coupon. Coupons after seeding were immediately exposed to increasing durations of high-intensity 405 nm light with an average irradiance of 110 mW cm^–2^ across the coupon surface. Following exposure, coupons were pressed onto a TSA surface for 5 seconds to recover the surviving bacteria from the seeded surface. This seeding and recovery process was also carried out for both the initial seeded population and the nonexposed control samples. These experiments were repeated in triplicate.

### 2.4. Plating and Enumeration

For the suspension exposure experiments, test and control samples were plated onto Nutrient Agar (*E. coli*,* S. enterica*, *S. sonnei*) (Oxoid, Basingstoke, UK), TSA (*L. monocytogenes*) (Oxoid, Basingstoke, UK), or Middlebrook 7H10 Agar with OADC enrichment media (*M. terrae*) (Becton Dickinson and Company, NJ, USA) plates. A WASP 2 spiral plater (Don Whitely Scientific Ltd, Shipley, UK) was used to plate out the samples, either as 50 *μ*L spiral plate or 100 *μ*L spread plate samples, with each sample being plated in at least triplicate. One millilitre pour plates were prepared if low counts were expected. Plates were incubated at 37°C for 24 hours, except *M. terrae* plates which were incubated at 37°C for 7 days, after which the plates were enumerated and results reported as CFU mL^–1^.

For the agar and inert surface exposure experiments, plates were incubated directly following exposure at 37°C for 24 hours with results reported as CFU per plate. Qualitative experiment plates were photographed using a Sony Cyber-shot DSC-T2 digital camera.

### 2.5. Statistical Analysis

Data points on each figure represent the mean results of two or more independent experiments, with each individual experimental data point being sampled in triplicate at least. Data points include the standard deviation and significant differences obtained from results. Significant differences were calculated at the 95% confidence interval using ANOVA (one way) with MINITAB software release 15.

## 3. Results

**Figure 2:**
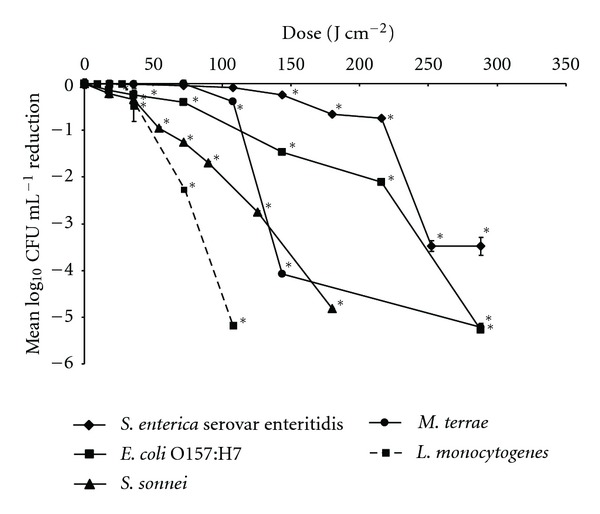
Inactivation of *S. sonnei, E. coli, S. enterica, L. monocytogenes,* and *M. terrae* in liquid suspension, by exposure to high-intensity 405 nm light of an irradiance of approximately 10 mW cm^–2^. Control samples remained constant throughout experimentation in all cases (data not shown). *Indicates where a light-exposed bacterial count was significantly different from the non-exposed control count (*P* ≤ 0.05 calculated at the 95% confidence interval).

Liquid bacterial suspensions were exposed to 405 nm light at an irradiance of 10 mW cm^–2^ for increasing time periods. From these values, the absolute dose applied in each experiment can be calculated using (2).  Figure 2 demonstrates the effect of 405 nm light exposure on suspensions of Gram negative bacteria—*E. coli*,—*S. enterica*, *S. sonnei*—and Gram positive bacteria—*L. monocytogenes* and *M. terrae*. *S. enteritidis* and *E. coli* were inactivated by 3.5-log_10_ CFU mL^–1^ and 5-log_10_ CFU mL^–1^, respectively, at 288 J cm^–2^, and *S. sonnei* was inactivated by 5-log_10_ CFU mL^–1^ at a lower dose of 180 J cm^–2^. *M. terrae* was inactivated by 4-5 log_10_ CFU mL^–1^ between a dose of 144 and 288 J cm^–2^, and *L. monocytogenes *was inactivated by 5-log_10_ CFU mL^–1^ at 108 J cm^–2^. It should be noted that the population densities of control samples for all five bacterial species remained constant throughout this series of experiments (data not shown).

Table 1 contains the results of exposing suspensions of *L. monocytogenes* to 108 J cm^–2^of 405 nm light, with the 108 J cm^–2^dose being applied in varying regimes. The results demonstrate that application of the dose, regardless of how it is applied (i.e., lower irradiance for longer exposure time or higher irradiance for shorter exposure time), yields very similar final populations (no statistical significant difference), in this case approximately a 5-log_10_ CFU mL^–1^ reduction in bacterial numbers.

In order to eliminate the possibility of inactivation being the result of heat transfer from the LED array during sample exposure, the temperatures of the bacterial suspensions were monitored during experimentation. Temperature readings (taken every 15 minutes) showed that the bacterial suspensions experienced minimal temperature changes during light exposure. Temperature was monitored over a 480-minute period (longest exposure time). The initial temperature of the suspensions was 26°C, which fluctuated ±1°C throughout the exposure period.

The inactivation achieved when bacterial-seeded agar plates were exposed to high-intensity 405 nm light (with an average irradiance of approximately 71 mW cm^–2^ across the plate) is shown in Table 2. With all four bacterial species tested, almost complete (100%) inactivation was achieved (<1 CFU/plate). *L. monocytogenes* was again inactivated at the fastest rate, with 100% reduction at an average dose of 128 J cm^–2^. *S. enterica*, *S. sonnei*, and *E. coli* were inactivated by 2.28 (100%), 2.10 (99.3%), and 2.18 (99.8%) log_10_ CFU/plate, respectively, at an average dose of 192 J cm^–2^.

**Figure 3:**
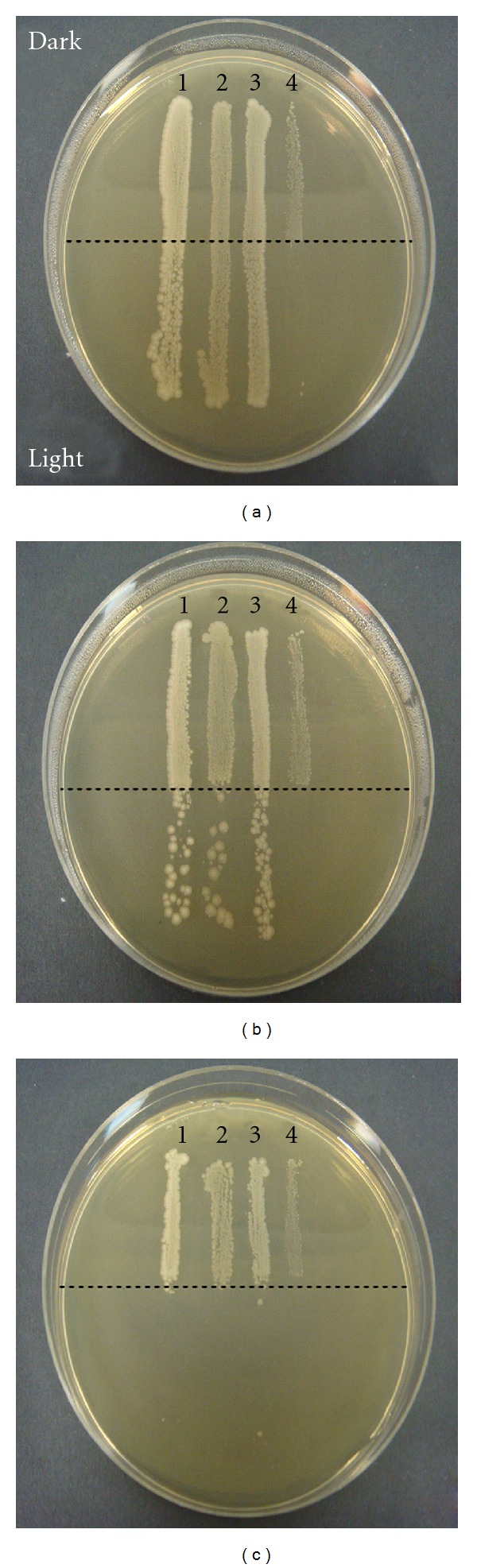
Qualitative representation of the bactericidal effect of high-intensity 405 nm light on (1) *E. coli*, (2) *S. sonnei, *(3) *S. enterica, *and (4) *L. monocytogenes. *Plates (a), (b), and (c) demonstrate the inactivating effect of 15, 30, and 45 minutes light exposure, respectively, on streaks of the foodborne pathogenic bacteria, with half of each plate being light exposed and the other half being kept in darkness to provide the equivalent non-light-exposed control.

Figure 3 shows a qualitative representation of the bactericidal effect of high-intensity 405 nm light on the four bacteria, *E. coli*, *S. sonnei, S. enterica, *and *L. monocytogenes*. The streaks of bacteria were light-exposed for 15, 30, and 45 minutes (Figures 3(a), 3(b), and 3(c), resp.). It can be seen that *L. monocytogenes *was the most susceptible and that by 45 minutes of 405 nm light exposure all of the tested species of bacteria were effectively inactivated.

The results of the 405 nm light inactivation of *S. enterica *and *L. monocytogenes* seeded onto acrylic and PVC surfaces are reported in Table 3. Both *S. enterica *and *L. monocytogenes* were inactivated more readily on the PVC surface using high-intensity 405 nm light than on acrylic surfaces. Interestingly, the Gram negative *S. enterica *was more rapidly inactivated than the Gram positive *L. monocytogenes* on both surfaces. *S. enterica *was inactivated by 2.19 log_10_ CFU/plate (100%) on PVC with an average dose of 50 J cm^–2^ and 1.63 log_10_ CFU/plate (98%) on acrylic with an average dose of 66 J cm^–2^. *L. monocytogenes* was inactivated by 0.90 log_10_ CFU/plate (90%) on PVC with an average dose of 50 J cm^–2^, and only 0.42 log_10_ CFU/plate (61%) on acrylic with an average dose of 66 J cm^–2^. When non-exposed control counts were compared to the counts of the initial seeded population it was found that between 70 and 80% of the seeded bacteria were nonrecoverable from acrylic and PVC surfaces after 7.5–10 minutes, likely as a result of inactivation by desiccation. Therefore, inactivation results were calculated as the reduction of light-exposed bacteria when compared to the respective non-exposed control and are reported as both reduction in log_10_ CFU/plate and % inactivation.

## 4. Discussion

This study has demonstrated that 405 nm light has a significant bactericidal effect on a number of important and taxonomically diverse bacterial pathogens. The current study set out with two aims: (1) to test the relative susceptibility of several diverse types of bacterial pathogens, including mycobacteria, which are among the most difficult of bacteria to inactivate using conventional decontamination techniques and (2) to determine the effectiveness of 405 nm light for inactivation of selected types of pathogens in both liquid suspensions and on surfaces.

The most resistant bacterium in liquid suspension was *S. enterica*, which was inactivated by 3.5-log_10_ CFU mL^–1^ at a dose of 288 J cm^–2^, around 2.5 times the dose required for 5-log_10_ CFU mL^–1^ inactivation of the least susceptible bacterium *L. monocytogenes *(108 J cm^–2^). *M. terrae* was inactivated by 4-5 log_10_ CFU mL^–1^ between 144 and 288 J cm^–2^, and *E. coli* and *S. sonnei* were inactivated by 5-log_10_ CFU mL^–1^ at 288 J cm^–2^ and 180 J cm^–2^, respectively.

*L. monocytogenes* also proved to be the most readily inactivated organism when seeded onto agar surfaces, with 100% inactivation achieved with an average dose of 128 J cm^–2^. The least susceptible microorganism, of those tested, in the agar surface exposure experiments appeared to be *S. sonnei* with a 2.10 log_10_ CFU/plate (99.3%) reduction in bacterial numbers achieved at an average dose of 192 J cm^–2^, however; in statistical tests this level of inactivation was not significantly different from the percentage inactivation rates achieved for *S. enterica *and *E. coli* at the same dose. It would be interesting to directly compare the susceptibility of the test bacteria exposed in liquid suspension to those seeded onto surfaces; however, the inactivation doses cannot be directly compared due to differences in the experimental arrangements and exposure conditions. Under suspension test conditions, uniform exposure of a well-mixed bacterial suspension was achieved, whereas with surface exposure tests, light irradiance varied over the surface, therefore requiring that an average power density/irradiance value be calculated in order to determine the corresponding dose values.

Reasons for the variable susceptibility of different bacteria to 405 nm light are as yet undetermined. Studies have reported that Gram positive species, in general, were more susceptible to 405 nm light inactivation than Gram negative species, which is generally consistent with the results obtained in the current study [21]. It is also theorised that the difference in inactivation kinetics may be due to organism-specific differences in porphyrin levels, porphyrin types, porphyrin wavelength absorption maxima, or as a consequence of the relatively short distance that singlet oxygen molecules can diffuse (~20 nm^3^ in solution) within cell structures [13, 21, 23]. In addition it has been speculated that less oxygen-tolerant bacterial species may be particularly susceptible to the effects of ROS as microorganisms such as some microaerophilic species have been found to possess fewer key oxidative regulators than most aerobes [24, 25]. Studies have shown blue light to be capable of inactivating the anaerobic oral pathogens *Prevotella*, *Porphyromonas,* and *Fusobacterium* as well as microaerophilic pathogens such as *Propionibacterium acnes* and *Helicobacter pylori *[15, 26–29]. However, inactivation results achieved with anaerobic/microaerophilic bacteria have not provided conclusive evidence as to whether oxygen-sensitive bacteria are any more susceptible than aerobes as many of these bacteria are also known to accumulate high levels of porphyrins.

The inactivation data for bacteria exposed in suspension in the present study can be compared with the 405 nm inactivation data obtained in other studies [21, 22]. *L. monocytogenes, *although much more readily inactivated than the microorganisms *S. sonnei, M. terrae*,* S. enterica,* and *E. coli*, was less susceptible than the majority of the medically significant Gram positive organisms (*Staphylococcus*, *Streptococcus*, *Clostridium* species) investigated by Maclean et al. [21]. All Gram negative organisms (*Acinetobacter*, *Proteus*, *Pseudomonas*, *Klebsiella,* and *Escherichia *species) tested by Maclean et al. [21] had inactivation rates comparable with the values found in the present study for *S. sonnei*, *E. coli, *and *S. enterica*. A notable exception to this is the Gram negative microaerophilic organism *Campylobacter jejuni*, investigated in a study by Murdoch et al. [22], which exhibited a particularly high sensitivity to 405 nm light. Interestingly, *M. terrae* showed similar inactivation kinetics to the Gram negative pathogens, particularly *S. sonnei. *Possible explanations for this difference in inactivation susceptibility could be that the unusual cell envelope, characteristic of mycobacteria, confers some resistance to 405 nm light penetration and/or that mycobacterial cell envelopes provide greater innate resistance to ROS given that mycobacteria species are capable of evading phagocytic oxidative burst damage [30].

A study by Bohrerova and Linden [31] into UV light inactivation showed *M. terrae* to be more resistant to the effects of the UV light than other representative Gram positive and Gram negative bacterial pathogens. The study demonstrated UV-light susceptibility in *M. terrae* and *M. tuberculosis* to be almost identical which corresponds well with studies by Maclean et al. [21], where they achieved similar inactivation kinetics between bacteria of the same genus when exposed to 405 nm light. The results of these studies also indicate the suitability of *M. terrae* as a reliable *M. tuberculosis* surrogate organism in light inactivation studies.

This study has demonstrated that exposure to high-intensity 405 nm light is capable of inactivating a variety of taxonomically diverse bacterial pathogens without the requirement for exogenous photosensitiser molecules. The bactericidal effect was demonstrated quantitatively in liquid suspension and both quantitatively and qualitatively on agar plates and inert surfaces. The inactivation process has been shown to be dose dependent; therefore, higher-intensity light-sources could achieve lethal doses in shorter time periods. The findings that the bacteria can be inactivated using high-intensity 405 nm light whilst seeded on both nutritious and inert surfaces is particularly significant for potential practical application within the food and healthcare industry, where cross-contamination from environmental contact surfaces and equipment is a problem. The fact that 405 nm light falls within the visible light range and does not require the containment conditions of harmful UV light potentially permits the continuous treatment of food contact areas in the presence of operator personnel, a uniquely advantageous feature. In order to evaluate the effectiveness of 405 nm LED arrays for practical applications, such as the continuous treatment of large surface areas, custom-designed light sources are required that can achieve a more uniform power density distribution, perhaps through the use of lenses. Future work to assess the treatment potential of 405 nm light, as well as further studies on the inactivation of bacterial biofilms, will also be important in order to fully assess the potential of this inactivation technology for applications within the food industry alongside other safety control methods.

## Figures and Tables

**Table 1:** The effect of applying a dose of 108 J cm^–2^ using three different power density/exposure time regimes on the 405 nm light inactivation of *L. monocytogenes* in suspension.

Power density (mW cm^–2^)	Exposure time (min)	Dose (J cm^–2^)	Log_10_ CFU mL^–1^ reduction
10	180	108	5.18* (SD ±0.2)
20	90	108	5.05* (SD ±0.0)
30	60	108	4.90* (SD ±0.4)

*Indicates where a light-exposed bacterial count was significantly different from the non-exposed control count (*P* ≤ 0.05 calculated at the 95% confidence interval).

SD: standard deviation of averaged results.

**Table 2:** Inactivation of bacteria seeded onto agar surfaces upon exposure to high-intensity 405 nm light for different times. Agar plates (9 cm diameter) were exposed to an average irradiance of 71 mW cm^–2^.

	Dose	*S. enterica *(Log_10_ CFU/plate ± SD)			*S. sonnei* (Log_10_ CFU/plate ± SD)			*E. coli* 0157:H7 (Log_10_ CFU/plate ± SD)			*L. monocytogenes* (Log_10_ CFU/plate ± SD)		
Exposure time (min)	(J cm^2^)	Non-light exposed	Light exposed	Log_10_ reduction (% reduction)	Non-light exposed	Light exposed	Log_10_ reduction (% reduction)	Non-light exposed	Light exposed	Log_10_ reduction (% reduction)	Non-light exposed	Light exposed	Log_10_ reduction (% reduction)
10	60	—	—	—	—	—	—	—	—	—	2.44 (±0.02)	2.37 (±0.03)	0.07* (15.3%)
15	90	2.38 (±0.03)	2.33 (±0.02)	0.05 (11.3%)	2.28 (±0.02)	1.82 (±0.12)	0.46* (64.4%)	2.32 (±0.02)	2.09 (±0.13)	0.23* (39.4%)	—	—	—
20	120	—	—	—	—	—	—	—	—	—	2.33 (±0.10)	1.65 (±0.30)	0.68* (76.4%)
30	180	2.33 (±0.01)	1.19 (±0.30)	1.14* (91.4%)	2.21 (±0.09)	0.93 (±0.40)	1.28* (93.3%)	2.27 (±0.03)	0.90 (±0.10)	1.37* (95.7%)	2.25 (±0.06)	0 (±0.00)	2.25* (100%)
45	270	2.28 (±0.02)	0 (±0.00)	2.28* (100%)	2.26 (±0.04)	0.16 (±0.30)	2.10* (99.3%)	2.18 (±0.02)	0 (±0.00)	2.18* (99.8%)	—	—	—

*Indicates where a light-exposed sample value was statistically significant from a non-exposed control value (*P* ≤ 0.05 calculated at the 95% confidence interval).

SD: standard deviation of averaged results.

**Table 3:** Inactivation of bacteria aerosolised onto PVC and acrylic surfaces upon exposure to 110 mW cm^–2^ of high-intensity 405 nm light.

			*S. enterica *(Log_10_ CFU/plate ± SD)			*L. monocytogenes* (Log_10_ CFU/plate ± SD)		
Surface material	Exposure time (min)	Dose J (cm)^−2^	Non-exposed	Light exposed	Log_10_ reduction (% reduction)	Non-exposed	Light exposed	Log_10_ reduction (% reduction)
PVC	2.5	15	2.54 (±0.10)	0.53 (±0.21)	2.01* (98%)	2.08 (±0.45)	1.41 (±0.18)	0.68* (78%)
	5	30	2.62 (±0.15)	0.72 (±0.40)	1.90* (99%)	1.93 (±0.28)	1.0 (±0.42)	0.93* (86%)
	7.5	45	2.19 (±0.03)	0 (±0.0)	2.19* (100%)	1.69 (±0.40)	0.79 (±0.1)	0.90* (90%)

Acrylic	2.5	15	2.76 (±0.13)	1.59 (±0.05)	1.18* (93%)	2.45 (±0.06)	2.21 (±0.22)	0.24 (36%)
	5	30	2.42 (±0.16)	1.18 (±0.31)	1.24* (93%)	2.37 (±0.06)	2.15 (±0.12)	0.22 (39%)
	7.5	45	2.66 (±0.1)	1.20 (±0.26)	1.46* (96%)	2.24 (±0.10)	2.02 (±0.17)	0.21 (36%)
	10	60	2.09 (±0.16)	0.46 (±0.15)	1.63* (98%)	2.18 (±0.16)	1.75 (±0.21)	0.42* (61%)

*Indicates where a light-exposed sample value was statistically significant from a non-exposed control value (*P* ≤ 0.05 calculated at the 95% confidence interval).

SD: standard deviation of averaged results.
